# Active and fast charge-state switching of single NV centres in diamond by in-plane Al-Schottky junctions

**DOI:** 10.3762/bjnano.7.165

**Published:** 2016-11-16

**Authors:** Christoph Schreyvogel, Vladimir Polyakov, Sina Burk, Helmut Fedder, Andrej Denisenko, Felipe Fávaro de Oliveira, Ralf Wunderlich, Jan Meijer, Verena Zuerbig, Jörg Wrachtrup, Christoph E Nebel

**Affiliations:** 1 Fraunhofer-Institute for Applied Solid State Physics (IAF), 79108 Freiburg, Germany; 2 3rd Institute of Physics, University of Stuttgart, 70569 Stuttgart, Germany; 3 Department of Physics and Geoscience, University of Leipzig, 04103 Leipzig, Germany; 4 Max Planck Institute for Solid State Research, 70569 Stuttgart, Germany

**Keywords:** active charge state control, diamond, fast charge state switching, NV centre, two-dimensional Schottky diode

## Abstract

In this paper, we demonstrate an active and fast control of the charge state and hence of the optical and electronic properties of single and near-surface nitrogen-vacancy centres (NV centres) in diamond. This active manipulation is achieved by using a two-dimensional Schottky-diode structure from diamond, i.e., by using aluminium as Schottky contact on a hydrogen terminated diamond surface. By changing the applied potential on the Schottky contact, we are able to actively switch single NV centres between all three charge states NV^+^, NV^0^ and NV^−^ on a timescale of 10 to 100 ns, corresponding to a switching frequency of 10–100 MHz. This switching frequency is much higher than the hyperfine interaction frequency between an electron spin (of NV^−^) and a nuclear spin (of ^15^N or ^13^C for example) of 2.66 kHz. This high-frequency charge state switching with a planar diode structure would open the door for many quantum optical applications such as a quantum computer with single NVs for quantum information processing as well as single ^13^C atoms for long-lifetime storage of quantum information. Furthermore, a control of spectral emission properties of single NVs as a single photon emitters – embedded in photonic structures for example – can be realized which would be vital for quantum communication and cryptography.

## Introduction

The nitrogen-vacancy centre (NV centre) in diamond is known to exist in at least three different charge states (NV^−^, NV^0^ and NV^+^ state). Compared to the positive and the neutral charge state, the negative state (NV^−^) shows superior properties for electron-spin related applications in quantum physics, namely a perfect photostability [1], an optical initialization and read-out of its ground state electron spin [2] and an optically detected magnetic resonance [3] at ambient conditions. Due to these outstanding properties it can be used for applications such as single-spin magnetometry [4], imaging in life science [5], quantum computing and quantum cryptography [6–7] at room temperature, i.e., without the need for cryogenic conditions.

For many quantum applications, near-surface NV^−^ centres are required in order to efficiently couple out the emitted photoluminescence or to increase its sensitivity to external magnetic fields. However, one drawback is that near-surface NV centres are strongly affected by surface defects, surface terminations and adsorbates. Hence, its charge state switches in an uncontrolled way between NV^−^, NV^0^ and presumably NV^+^ [8–10].

In addition, for realizing quantum computing applications such as quantum information processing with the electron spin of the NV^−^ centre and quantum information storage with the nuclear spin of the ^15^N or ^13^C atom, it is necessary not only to stabilize the NV^−^ state but also to switch actively between NV^−^ (bright state, with electron spin) and NV^+^ (dark state, without electron spin). It was recently shown that a transfer of the spin state from an electron spin to a nuclear spin leads to very large T_2_ times [11] which is required for quantum information storage as well as for conserving the entanglement with high fidelity [12]. Therefore, an important requirement for realizing quantum optical applications with single NV centres is not only an active charge state control but also a fast switching between all three charge states NV^−^, NV^0^ and NV^+^.

To understand the importance of fast switching, we will now consider one application example of a quantum computer consisting of NV centres and ^13^C atoms in the diamond crystal, arranged as quantum registers. The electron spin of an NV^−^ centre can be used for initialising, manipulating and processing of quantum information as well as entanglement of its spin state with other NVs as quantum bits. The nuclear spin of a ^13^C atom can be used for storage of quantum information with a long lifetime. In order to achieve a long lifetime of stored quantum information with high fidelity we have – after transfer of the quantum state from the electron to the nuclear spin – to decouple the electron spin from the nuclear spin. For this, the NV centre need to be switched from NV^−^ (optically active, with electron spin) to NV^+^ (optically inactive, without electron spin). The stored quantum information can be read out optically by transferring the spin state from nuclear spin back to the electron spin. For this, the NV centre need to be switched back from NV^+^ (optically inactive, without electron spin) to NV^−^ (optically active, with electron spin). In both cases the switching speed should be faster than the rate of hyperfine interaction between an electron and nuclear spin of A_//_ = 2.66 kHz [13] to avoid any negative interplay between both spins during switching processes.

In order to control and to switch actively the charge state of a single NV centre, we have to control the position of the Fermi-level relative to charge transition levels NV^+/0^ and NV^0/−^ of the centre within the bandgap of diamond. The charge transition level of a defect centre is defined as a level at which the centre takes up or loses an electron when the Fermi level crosses this level. A control of the Fermi-level position can be performed either passively by the chemical control via surface termination with oxygen [8–10] or fluorine [14] or actively by an electrical control with structures such as a solution-gated field effect transistor [15], a pin-diode [16] or an in-plane gate transistor [17]. However, the above-mentioned passive control is static, i.e., the charge state cannot be switched on demand. In addition, with the above-mentioned techniques of electrical control it enables a control and switching between only the two (optical active) charge states NV^−^ and NV^0^.

In this work, we demonstrate for the first time an active control and a fast switching of the charge state of single NV centres between all three charge states (NV^−^, NV^0^ and NV^+^) by using a two-dimensional diamond Schottky-diode structure on a hydrogen-terminated (H-terminated) diamond surface. A H-terminated diamond surface exhibits a two-dimensional hole channel a few nanometres below the surface and an aluminium (Al) contact forms a two-dimensional Schottky junction (i.e., in planar configuration) with the p-type surface conductive channel inducing a lateral hole depletion layer below and next to the Al contact [18–19]. By applying potentials on the Al-Schottky contact, the applied electric field in the depletion region of the Schottky junction induces a band bending modulation which shifts the Fermi-level over NV charge transition level and the charge state of this centre is switched in an active and controlled way [20–21].

This paper is structured as follows: First as an introduction, we give a very short summary of the fabrication of the Schottky diode and the results published in a previous paper showing that spectral properties of the emitted photoluminescence of single NV centres is changed upon applying reverse bias potentials on the Al-Schottky contact indicating an active switching from the nonfluorescent NV^+^ state to the fluorescent state NV^0^ or NV^−^ and vice versa [21]. Then we will show the latest results on the fast switching of the NV charge state. Details on the diamond growth, fabrication of Schottky-diode and experimental setup for optical detection are described in [20–21].

## Sample preparation

An Ib (100) 3 × 3 × 0.3 mm^3^ diamond plate from Element Six Ltd. was used as a substrate and a 300 µm thick intrinsic epi-layer with high purity (i.e., without optically active defect centres such as NV centres) was grown homoepitaxially onto the substrate using an ellipsoidal shaped microwave plasma-enhanced chemical vapour deposition (MWPECVD) reactor [22]. After growth, the substrate was removed by laser cutting, the epi-layer mechanically polished to get a smooth surface [23] and cleaned wet chemically which results in oxygen-termination (O-termination) of the surface.

In the next step, the freestanding intrinsic diamond sample was implanted with nitrogen ions with implantation energy of 5 keV corresponding to a mean depth of 8.2 nm according to a SRIM (stopping and range of ions in matter) simulation [24]. The implanted dose of nitrogen was 10^8^ ions/cm^2^ covering homogenously the surface. The sample was annealed for 2 h at 800 °C in vacuum to create NV centres. The formation efficiency is about 1–10% [25–26] which leads to an NV centre sheet density in of [NV] = 10^6^–10^7^ cm^−2^.

We used confocal microscopy with an excitation laser of 520 nm to detect the NV centre photoluminescence in the depletion region of the Schottky junction. Distinct spectral characteristics can be used to identify the charge state: the NV^+^ centre does not show fluorescence upon optical excitation, whereas NV^0^ and NV^−^ show a zero phonon line at 575 nm at 637 nm, respectively, with their corresponding phonon side bands [8].

**Figure 1:**
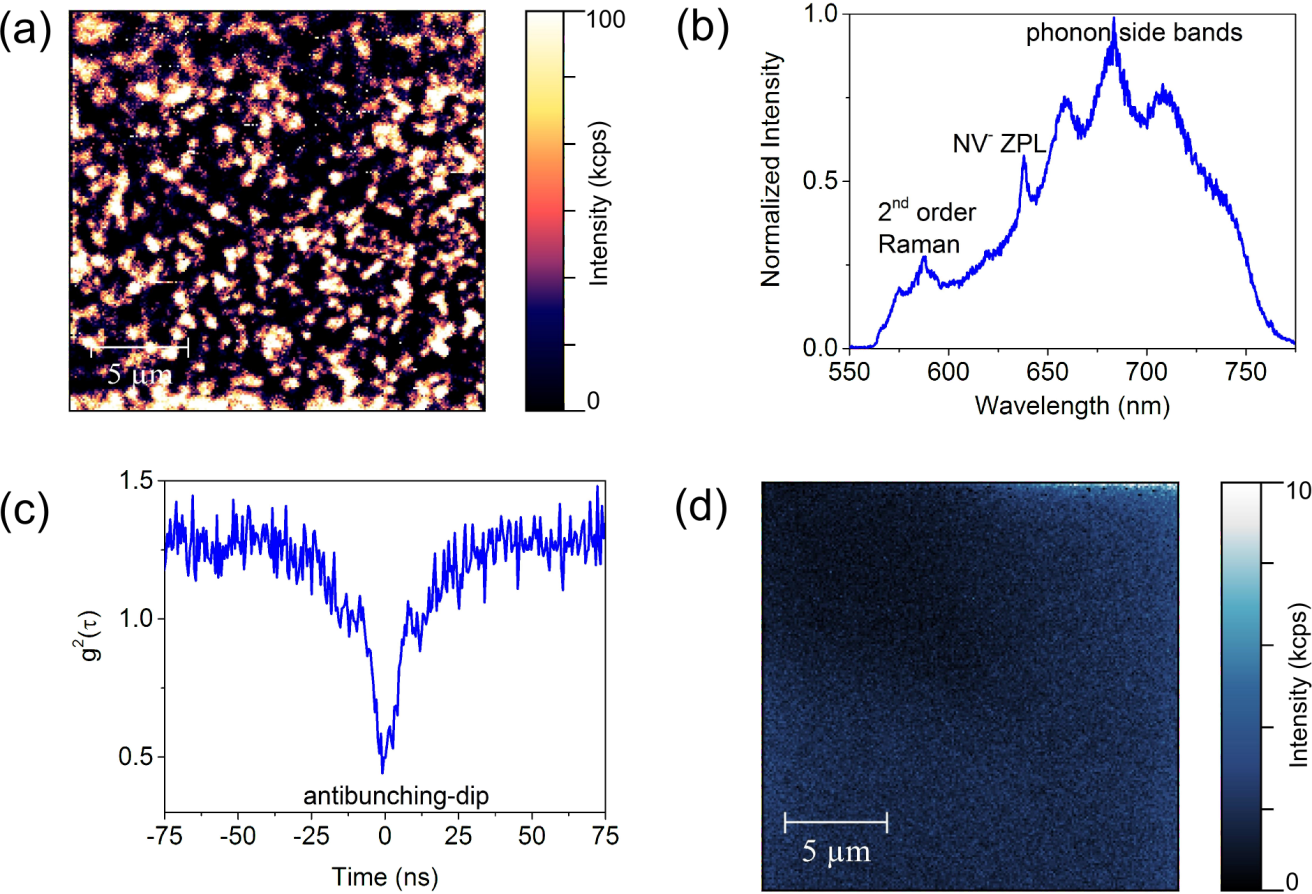
Formation and quenching of NV centres. (a) PL-intensity mapping performed on the diamond surface which shows bright spots. These bright spots are single NV^−^ centres according to the corresponding (b) PL-spectrum and (c) second-order photon autocorrelation measurement performed with a Hanbury–Brown and Twiss interferometer setup showing a clear antibunching dip indicating single photon emission. (d) After H-termination of the diamond surface the NV-emission is quenched due to the conversion from the fluorescent state NV^−^ to NV^+^, i.e., only a PL-background of the surface is visible.

A PL-intensity mapping performed on the surface shows bright spots originating from NV luminescence (Figure 1a). An analysing of PL-mapping images of mapping size of 20 × 20 µm^2^ each reveals an average NV density of approximately 10^7^ cm^−2^. This would correspond to a formation efficiency of approximately 10% which is in agreement with formation efficiency values reported in literature [25–26]. The bright spots in the mapping images were identified as single NV^−^ centres according to a measurement of the spectrum (Figure 1b) and the second-order photon autocorrelation with a Hanbury–Brown and Twiss interferometer setup [1,27–28], which shows a clear antibunching dip at τ = 0 s (Figure 1c).

Before the realisation of a two-dimensional Schottky diode, the diamond surface was hydrogen-terminated (H-terminated) by applying a pure H-plasma in the MWPECVD reactor which results in the formation of a two-dimensional hole accumulation layer a few nanometres below the surface [18]. Due to the interaction with the two-dimensional hole channel, NV centres are quenched, i.e., switched to the nonfluorescent state NV^+^ as proved by PL-intensity mappings which show no bright spots (Figure 1d).

**Figure 2:**
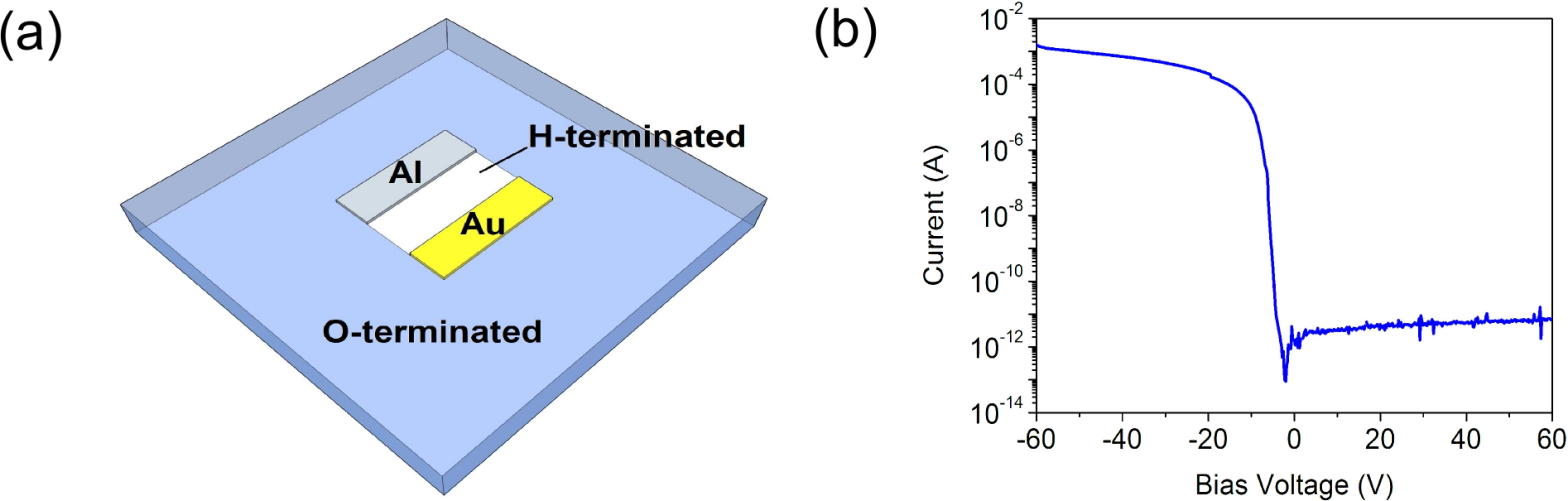
In-plane Schottky diode from diamond. (a) Schematic figure of an in-plane Al Schottky diode on an H-terminated diamond surface. (b) The current–voltage properties of the fabricated in-plane Al-Schottky diode measured at ambient conditions.

For fabricating a two-dimensional Schottky diode structure, aluminium (Al) and gold (Au) contacts with a thickness of 200 nm each were deposited onto the diamond surface using photolithography with subsequent thermal evaporation of the metals. Al is a Schottky contact showing a barrier height of 570 meV and Au is an Ohmic contact [18]. The contacts exhibit dimensions of 1 mm × 300 µm and were separated from each other by 400 µm. In order to have a defined conductive channel between the Al and Au contacts, the channel region including the contacts were protected with a photoresist and then the whole diamond surface surrounding the channel area were O-terminated via exposition to oxygen plasma. After lift-off process of the photoresist, the fabrication of the in-plane diamond Schottky diode is finished, which is shown schematically in Figure 2a. The current–voltage characteristic of this diode, measured at ambient conditions, shows very good Schottky properties (Figure 2b).

## Results and Discussion

### Active charge-state switching

**Figure 3:**
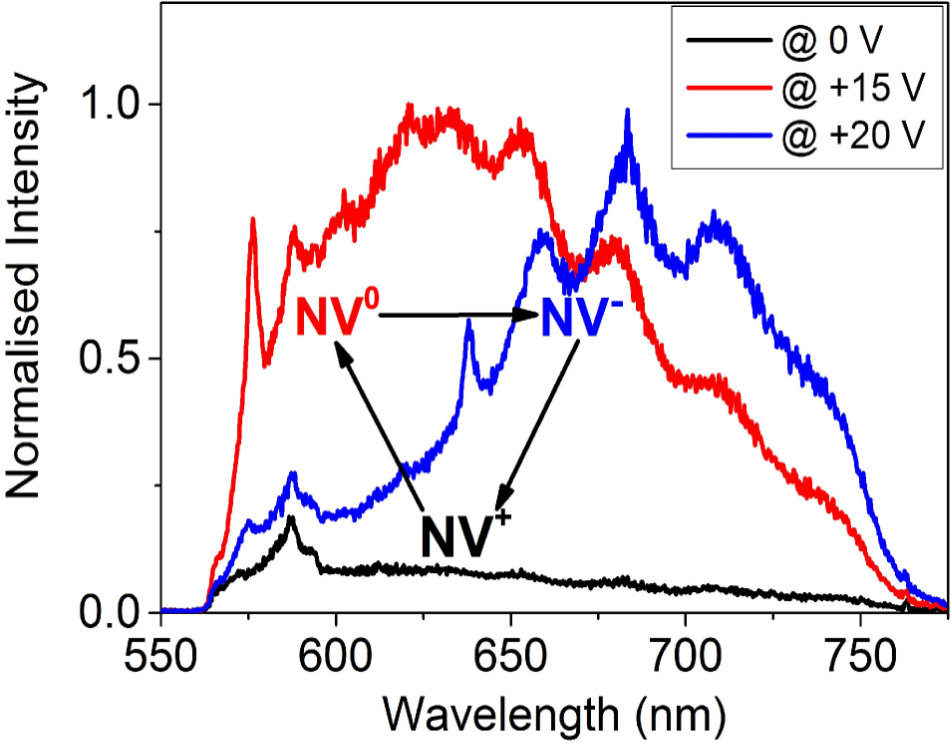
Active charge state control of a single NV centre. At zero bias there is only a PL-background of the diamond surface (black spectrum) since the NV centre is in the nonfluorescent NV^+^ state due to the interaction with the two-dimensional hole accumulation layer. Upon applying a reverse bias potential of +15 V, we detect a spectrum of NV^0^ emission (red spectrum). Upon applying a higher reverse bias potential (+20 V) we detect a spectrum of NV^−^ emission (blue spectrum).

As already published elsewhere, via analyzing PL-spectra at different potentials applied on the Schottky contact, we could demonstrate an active control of the charge state of a single NV centre in the depletion region of the in-plane Schottky junction [21]. At zero bias there is only a PL-background of the diamond surface (black spectrum in Figure 3), since the NV centre is in the NV^+^ state which shows no fluorescence. Upon applying a reverse bias voltage of +15 V, the measured spectrum clearly shows NV^0^ emission with the characteristic zero phonon line at 575 nm and its phonon side band (red spectrum in Figure 3). By increasing the reverse bias voltage to +20 V, the measured spectrum shows NV^−^ emission with the characteristic zero phonon line at 637 nm and its phonon side band (blue spectrum in Figure 3). After switching off the applied bias voltage, the recorded PL-spectrum revealed only the previously measured background spectrum of the diamond surface (black spectrum of Figure 3), which indicates the NV centre to be in the nonfluorescent NV^+^ state.

### Fast charge-state switching

**Figure 4:**
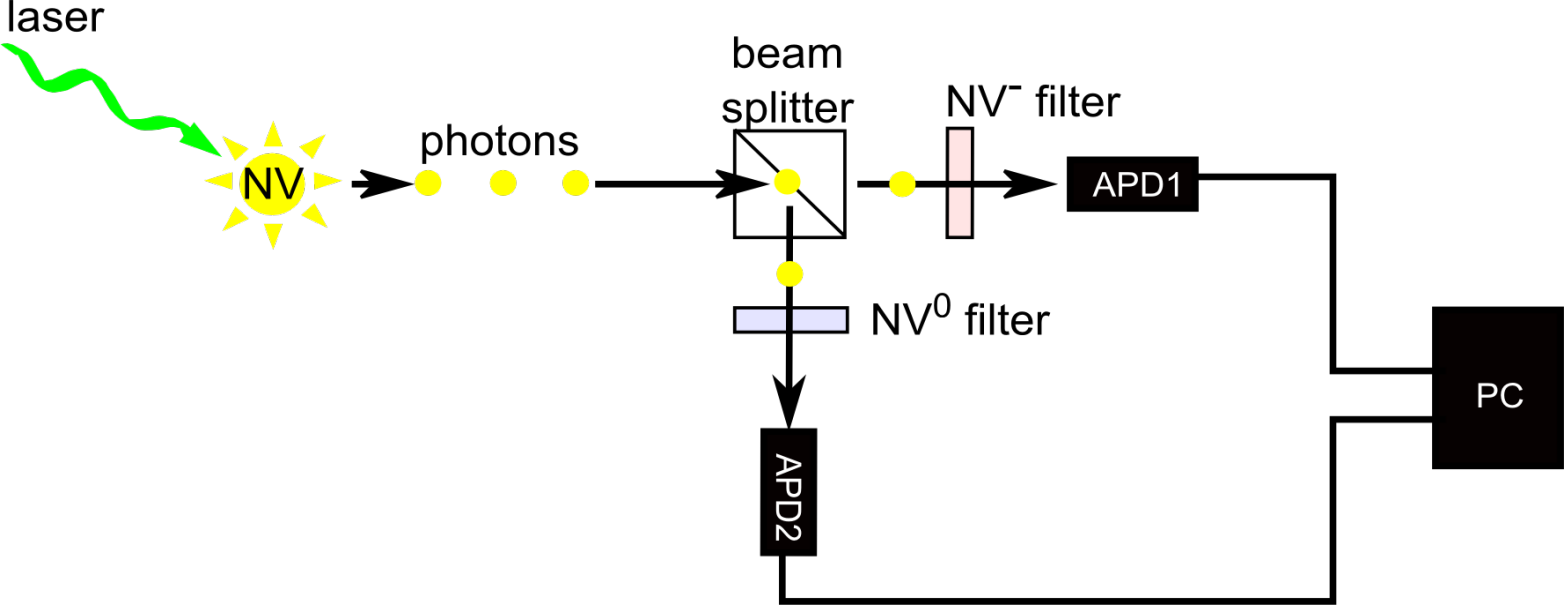
Schematic setup for time-resolved measurement of NV-intensity. To record the time evolution of the intensity of NV^−^ and NV^0^ emission during switching between NV^−^ or NV^0^ and NV^+^, the single NV centre was illuminated continuously with a 520 nm laser during the switching cycles and the emitted NV-photoluminescence was routed via a beam splitter to two avalanche photo diodes with corresponding NV filters in front of them.

In order to answer the question of how fast the charge state of a single NV centre can be switched with the two-dimensional diamond Schottky diode and to understand the underlying switching mechanisms, we performed a time-resolved measurement of NV-intensity during switching between the fluorescent (NV^−^ and NV^0^) and nonfluorescent (NV^+^) states. To record the time evolution of the NV intensity during switching between NV^−^ or NV^0^ and NV^+^, the single NV centre was illuminated continuously with a 520 nm laser and the emitted NV-photoluminescence was routed via a beam splitter to two avalanche photo diodes (APD) with corresponding NV (NV^0^ and NV^−^) filters in front of them. The time resolution for detecting the intensity change by an avalanche photo diode is in the range of 1–10 ns. A schematic of this measurement setup is shown in Figure 4.

For switching the applied potential on the Schottky diode, we used a FPGA-based pulse generator in combination with an amplifier. For switching between NV^0^ ↔ NV^+^ or NV^−^ ↔ NV^+^, a rectangle pulse of amplitude ±15 V or ±20 V with a period of 4 µs was applied. The voltage ramping for switching between ±20 V takes place within 100 ns. Thus, a switching from NV^−^ (+20 V) to NV^+^ (<+15 V) and vice versa will take place theoretically within 10–15 ns.

**Figure 5:**
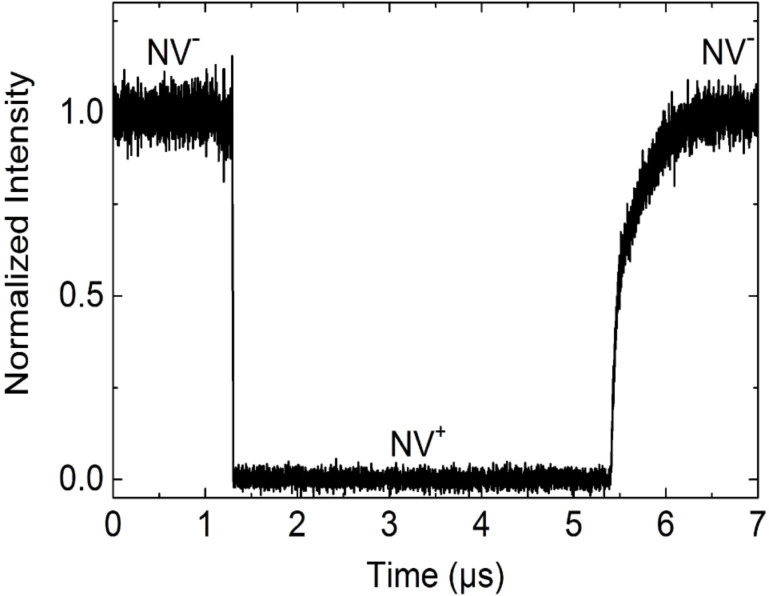
Fast charge state switching. The result of the time-resolved NV-intensity measurement shows that switching from NV^−^ to NV^+^ takes place much faster than switching from NV^+^ to NV^−^. Since in this case we used an NV^−^ filter in front of an ADP, the maximum and the minimum of the detected intensity corresponds to the NV^−^ and NV^+^ state respectively.

**Figure 6:**
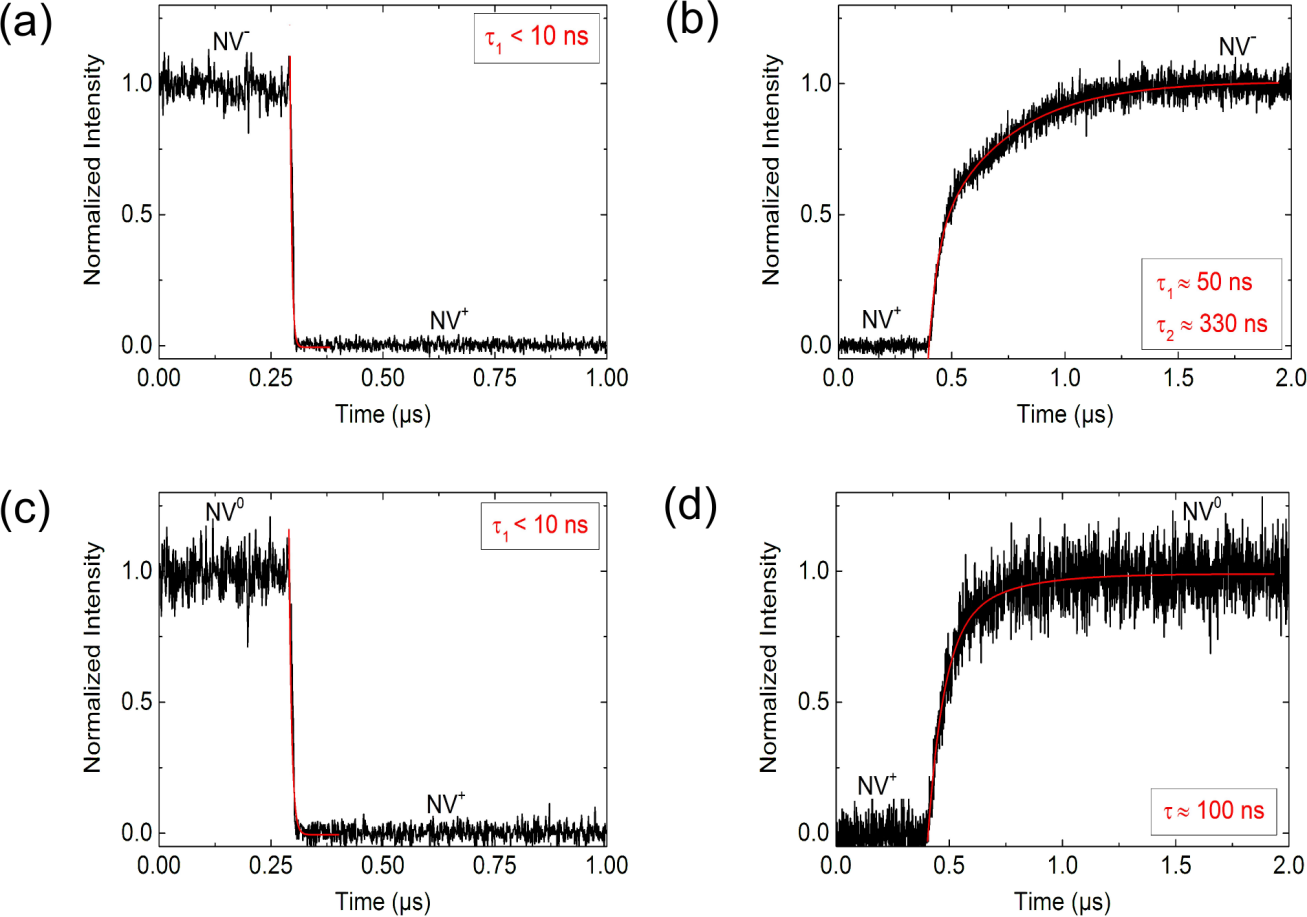
Time constant for charge state switching. Time constants deduced from fitting an exponential function to the decay and rise of NV-intensity for the transitions (a) NV^−^ → NV^+^ (b) NV^+^ → NV^−^ (c) NV^0^ → NV^+^ as well as (d) NV^+^ → NV^0^. Since the time resolution of this measurement is in the range of 1–10 ns, the deduced time constants in (a) and (c) are upper limits. According to the measured time constants, a switching rate between 1 MHz (charging NV) and 100 MHz (discharging NV) seems to be possible. This is much faster than the rate of hyperfine interaction between an electron and nuclear spin of A_//_ = 2.66 kHz.

The result of the time-resolved NV-intensity measurement in Figure 5 shows that switching from NV^−^ to NV^+^ generally takes place much faster than switching from NV^+^ to NV^−^. In Figure 6, all results of a time-resolved intensity measurement for switching between the states NV^−^ ↔ NV^+^ or NV^0^ ↔ NV^+^ are summarised.

From fitting the following exponential function to the decrease and increase of the NV-intensity, we could deduce the time constants τ_1_ and τ_2_:


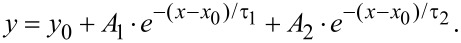


A summary of measured time constants for switching a single NV centre between the fluorescent and nonfluorescent states as well as the corresponding calculated maximum (theoretical) switching frequencies is given in Table 1. According to the measured time constants, a switching frequency between approximately 1 MHz (charging NV) and 100 MHz (discharging NV) seems to be possible. This is much faster than the rate of hyperfine interaction between an electron and nuclear spin of A_//_ = 2.66 kHz [13].

**Table 1:** Summary of measured switching speed. Measured time constants for switching a single NV centre between the fluorescent and non-fluorescent states as well as the corresponding calculated maximum (theoretical) switching rates.

NV-transition	time constant	max. switching frequency

NV^−^ → NV^+^	τ < 10 ns	>100 MHz
NV^+^ → NV^-^	τ_1_ ≈ 50 ns, τ_2_ ≈ 330 ns	≈1 MHz
NV^0^ → NV^+^	τ < 10 ns	>100 MHz
NV^+^ → NV^0^	τ ≈ 100 ns	≈10 MHz

With the help of the Software ATLAS from Silvaco Inc., we simulated this NV charge state switching by the Al-gate of the two-dimensional diamond Schottky diode to get a deeper understanding of the underlying mechanisms. The NV centre is switched from NV^−^ → NV^0^ → NV^+^ or switched from NV^+^ → NV^0^ → NV^−^ via an interaction with the two-dimensional hole channel, i.e., the centre changes its charge state upon capturing holes from the channel (non-radiative transition, Shockley–Read–Hall mechanism) or thermally emitting holes into the channel. Details of the results of charge state switching simulations and the deduced charge state switching mechanisms will be published in an upcoming paper.

**Figure 7:**
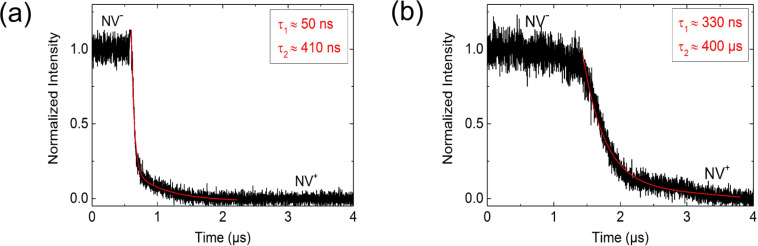
Degradation effect. After several 1000 switching cycles, the measured time constants for discharging NV^−^ increases to (a) approximately 500 ns. After additional several 1000 switching cycles the measured time constant (b) increases by one order of magnitude to approximately 500 µs. This indicates a degradation effect of the diode properties. The reasons for the observed degradation is explained in the text. Since the time constant increases above the time resolution of the measurement setup, we can now measure two time constants for the process of discharging NV^−^ to NV^+^, indicating a two-step process.

After several 1000 switching cycles, the measured time constants for discharging NV^−^ increases more than one order of magnitude as shown in Figure 7, indicating a degradation effect of the diode properties.

**Figure 8:**
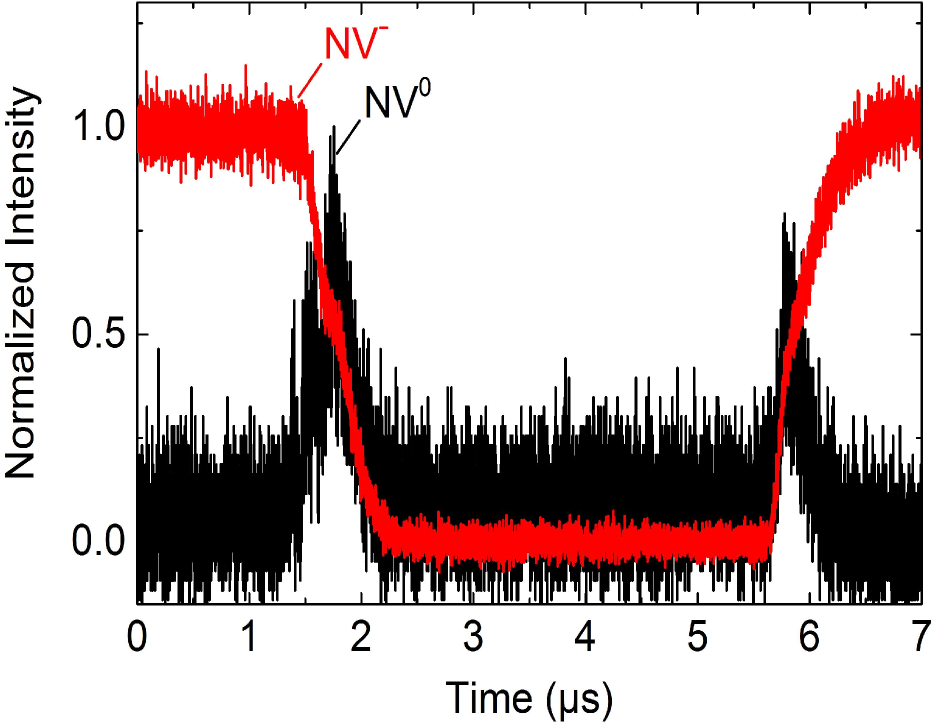
Simultaneous measurement of NV^−^ and NV^0^ intensity. A time-resolved measurement of NV^−^ (red curve) and NV^0^ (black curve) intensity simultaneously during switching between NV^−^ and NV^+^ shows that the charging and discharging process is a two-step process with NV^0^ as an intermediate state.

Since the time constant increases above the time resolution of the measurement, we can now measure two time constants for the process of discharging NV^−^ → NV^+^ like for the process of charging NV^+^ → NV^−^, too. From this, we can conclude that the transition NV^−^ ↔ NV^+^ is a two-step process with NV^0^ as an intermediate state. This assumption is supported by the result of the simultaneous measurement of NV^−^ and NV^0^ intensity during switching between NV^−^ and NV^+^ (Figure 8). It shows the occurrence of NV^0^ emission during the transition between the negative and positive charge state.

The larger time constant of the second step in the two-step process of charging/discharging the NV centre (see Figure 6b and Figure 7) can be explained by the change of the charge carrier capture cross section of the NV centre. Generally, it is well known that the charge carrier capture cross section is larger for a charged defect centre (here: NV^−^ or NV^+^) compared to a neutral defect centre (here: NV^0^).

The observed degradation of NV-switching in Figure 7 and diode properties respectively after many switching cycles is due to the interaction of the H-terminated diamond surface with air, particularly with oxygen (O_2_) and ozone (O_3_) [29]. This interaction is catalysed by illumination of the surface with laser light and application of potential on the diode. Due to this reaction the diamond surface is locally O-terminated leading to an irreversible disappearance of the two-dimensional hole channel. This assumption is supported by the fact that after many thousands of switching cycles, it is not possible any more to switch the NV charge state actively. Since the stability of the two-dimensional hole channel is influenced by the interaction with the atmosphere, the H-terminated diamond surface need to be passivated to get a stable device operation and NV manipulation. One passivation method was suggested by Hiraiwa et al. by covering the surface with an Al_2_O_3_ film using an atomic-layer-deposition (ALD) method with an H_2_O oxidant at 450 °C [29]. They could show that this film does not destroy the C–H bonds as well as the atmospheric adsorbate layer on top of the H-terminated surface which is required for the generation of a two-dimensional hole channel. In this way, the hole channel and thus the diode properties are preserved to temperatures up to 550 °C in air. Chemically, this stability is supported by the fact that both the thermal decomposition of C–H bonds and the reaction between C–H bonds and H_2_O in the Al_2_O_3_ layer are endothermic processes. This passivation method for preserving the properties of the two-dimensional hole channel and thus of the two-dimensional Al-Schottky diode for active manipulation of single NV centres will be evaluated in the upcoming experiments.

### Summary and Conclusion

In this paper, we presented the experimental results of active control and high-frequency switching of the charge state of single and near-surface NV centres with a two-dimensional Al-Schottky diode from diamond. By applying potentials on the Al-Schottky contact and simultaneous illumination of an NV centre with a 520 nm cw laser light, we can actively switch single NV centres between the charge states NV^+^, NV^0^ and NV^−^. This is due to the field-induced band bending modulation in the depletion region of the Schottky junction which shifts the Fermi-level over NV charge transition levels in such a way that the charge state of a single NV centre and thus its electrical and optical properties are tuned.

The measured switching frequency for the transition NV^−^/NV^0^ → NV^+^ and NV^+^ → NV^0^/NV^−^ is 100 MHz and 1 MHz respectively. The frequencies of combined optical and electrical switching are three orders of magnitude higher than the rate of hyperfine interaction between an electron and nuclear spin of A_//_ = 2.66 kHz. Furthermore, they are higher compared to the frequencies achieved by other technologies. For example, for pure optically induced switching between NV^0^ and NV^−^, the maximum rate is 1 MHz, due to two-photon-absorption processes [30–31]. And for a pure electrically induced switching between NV^0^ and NV^−^ in the intrinsic layer of a diamond pin diode, the maximum rate is 0.7 MHz [13,30], due to a larger capacitance of the three-dimensional diode. In addition – contrary to the above mentioned alternative technologies – with a two-dimensional Al-Schottky diode, presented in this paper, it is possible to switch between all three charge states NV^+^, NV^0^ and NV^−^, since it enables a shift of the Fermi-level over both NV charge transition levels NV^+/0^ and NV^0/−^. From simulations we could deduce that holes from the two-dimensional channel play a dominant role in switching the NV charge state, i.e., the centre changes its charge state upon capturing holes from the channel (non-radiative transition, Shockley–Read–Hall mechanism) or thermally emitting holes into the channel, depending on the direction of the bias switching.

This high-frequency charge state switching via a planar diode structure would open the door for many quantum optical applications such as a quantum registers with single NVs for initialization, processing and read-out as well as single ^13^C atoms for storage of quantum information. This could be realised with a suitable patterning of the Al-gates, such that single NV centres could be addressed individually. Furthermore, a control of the spectral emission properties of NVs as a single photon emitters – embedded in photonic structures for example – could be realized which would be vital for quantum communication and cryptography.
